# Preliminary evidence that eye appearance in parrots (Psittaciformes) co-varies with latitude and altitude

**DOI:** 10.1038/s41598-024-63599-3

**Published:** 2024-06-04

**Authors:** Elif Duran, Juan Olvido Perea-García, Diede Piepenbrock, Celine Veefkind, Mariska E. Kret, Jorg J. M. Massen

**Affiliations:** 1 Department of Psychology, Izmir University of Economics, Izmir, Turkey; 2 Institute of Psychology, Cognitive Psychology Unit, Leiden University, Wassenaarseweg 52, Leiden, 2333 AK The Netherlands; 3 Animal Behaviour and Cognition, Department of Biology, Utrecht University, Utrecht, The Netherlands; 4 Leiden Institute of Brain and Cognition (LIBC), Leiden, The Netherlands; 5 Vogelpark Avifauna, Alphen Aan Den Rijn, The Netherlands

**Keywords:** Evolution, Biodiversity, Ecology, Evolutionary ecology

## Abstract

External eye appearance in avian taxa has been proposed to be driven by social and ecological functions. Recent research in primates suggests, instead, that, photoprotective functions are important drivers of external eye appearance. Using similar methods, we examined the variation in external eye appearance of 132 parrot species (Psittaciformes) in relation to their ecology and sociality. Breeding systems, flock size and sexual dimorphism, as well as species’ latitude and maximum living altitude, and estimated UV-B incidence in species’ ranges were used to explore the contribution of social and ecological factors in driving external eye appearance. We measured the hue and brightness of visible parts of the eye and the difference in measurements of brightness between adjacent parts of the eye. We found no link between social variables and our measurements. We did, however, find a negative association between the brightness of the inner part of the iris and latitude and altitude. Darker inner irises were more prevalent farther away from the equator and for those species living at higher altitudes. We found no link between UV-B and brightness measurements of the iris, or tissue surrounding the eye. We speculate that these results are consistent with an adaptation for visual functions. While preliminary, these results suggest that external eye appearance in parrots is influenced by ecological, but not social factors.

## Introduction

Variation in external eye appearance has been inspected in many different animal taxa, but still little is known about its adaptive value. Research has primarily focused on primates^1–4^, due to the notion that human eyes are uniquely adapted for signaling functions^5^. Nevertheless, studies on the topic have also examined such broadly distributed groups as fish^6^, frogs^7^, and birds^8–13^. Apart from signaling functions, previous research has investigated the notion that pigmentation patterns in and around the eye serve photo-regulatory functions, either to enhance visual functions in uneven lighting^14^ or to protect the eye from damaging doses of ultraviolet (UV) radiation and, possibly, bacterial degradation^3,15^. In a large sample of anthropoid primate species (N = 77)^3^, found iris color to change depending on the distance of the species’ distribution from the equator. Fur, hair, skin, and feathers can provide protection against ultraviolet radiation, especially as they become darker closer to the equator^16–19^. Similarly, species closer to the equator have more pigmented conjunctivae, supporting the idea of a photoprotective role for ocular pigmentation^3^.

The shape of the palpebral fissure (ie., the opening between the eyelids and amount of eyeball this exposes) has also been investigated with regards to allometric scaling rules^3,5,20^. The amount of exposed eyeball (measured as the sclera size index or SSI^5^) increases with body weight^3,18^. SSI is calculated by dividing the width of the exposed eyeball by the diameter of the iris^21^. Especially in larger animals, it is less energetically demanding to move the eyes compared to the head, which is more massive. This is because the relative growth of the eyes to body size is smaller than the relative growth of the eyes to body weight. In other words, the relative difference in mass between eyes and head is much larger in larger animals, with eyes being much lighter than the head^20^. While the allometric scaling relationship between eye and head mass should hold across vertebrate taxa, it may be less applicable to birds because their skeletons are lighter than those of primates^22–24^. In primates, head and body mass increase proportionally to the cube of body height. However, moving them requires an increase in force of only the square of body height, because this force depends on the size of the muscle cross-section^20^. Because avian skulls are relatively lighter than those of mammals, they may not be as energetically costly to move, thus not driving the evolution of highly movable eyeballs as may have occurred in primates. Supporting this hypothesized relaxed scaling between eye and head mass is the observation that, even though birds can move their eyeballs, they largely rely on head movements to reorient their attention^25^. While recent studies have proposed a link between SSI and pigmentation around the iris in mammals^25^, we do not explore this link here because our ocular measurements do not include tissues adjacent to the iris.

In a review about the functions of external eye appearance in birds, Corbet et al.^26^ also distinguished between proposals focused on ecological (“survival-based”) and signaling functions. For ecological functions Pasarotto et al.^27^ proposed that nocturnal owls tend to have darker irises compared to other owl species with diurnal and crepuscular habits to avoid detection by prey while hunting. Similarly, Davidson et al.^28^ found that birds with non-cavity nests evolved to have darker eye features compared to cavity nesters, who had more conspicuous eyes. Alternative results, however, suggest that dark pigmentation near the pupil has a role in reducing glare and improving vision in *Turnix* species, thus suggesting anti-glare functions^29^. Among signaling functions, for example, is Davidson et al.’s^9^ proposal that the conspicuous eyes of jackdaws can be seen outside of the nest, so it is a useful cue to prevent conflict with other birds that may be interested in nesting there. Furthermore, a growing number of studies report that birds can voluntarily change their pupil size during social interaction associated with agonistic behavior, happiness, or excitement^30,31^. One study shows that, in Japanese quails, increases in pupil size can be observed during pleasant behavioral sequences like dustbathing^32^. Unlike the smooth muscle fibers in the iris of mammals, those of birds are predominantly striated^33^, suggesting that avian pupil size changes can be under voluntary control. Numerous lay reports also note the propensity of *Psittacidae* (parrots) to change pupil size rapidly as part of visually striking displays, known as “eye pinning” or “eye flashing”, which is also associated with agonistic behavior, happiness, or excitement^30,31,34,35^. However, findings pertaining to the social functions of iris colour in birds are generally lacking. In short, some evidence suggests communicative functions for the external eye appearance of birds, either as part of displays or by enabling the inference of internal states and emotions, but these suggestions have been sparsely investigated.

**Figure 1:**
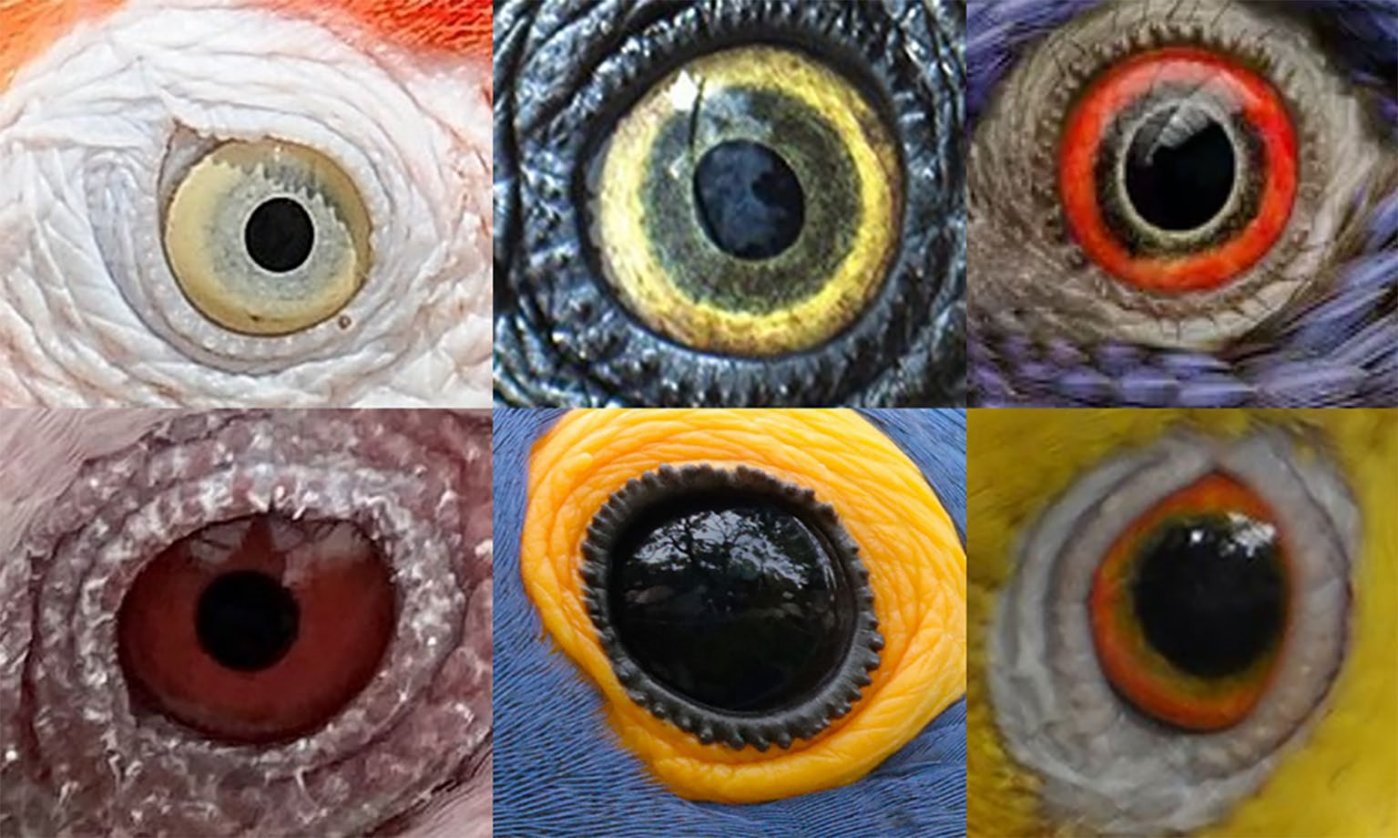
Coloration of six different species from left to right, top to bottom: *Ara macao*, *Deroptyus accipitrinus*, *Trichoglossus haematodus*, *Eolophus roseicapilla*, *Anodorhynchus hyacinthinus*, *Alipiopsitta xanthops*, respectively.

Among birds, parrots (Psittaciformes) are an interesting taxonomic group to test hypotheses about the relative contribution of social and environmental selection pressures in shaping the appearance of the eye. Most of the species within the Psittaciformes order (i.e., parrots) do not have visible sclera, however, iris coloration is extremely diverse from bright to dark colors, with species showing stark contrast between the inner (proximal to the pupil) and outer (distal to the pupil) portion of the iris (see Fig. 1). In addition, parrots have: (I) a wide range of body sizes, from 14 g (*Micropsitta pusio*) to 2250 g (*Strigops habroptilus*^36^ in our sample, which makes them an interesting system to explore the relationship between body size and exposure of the eyeball, as was found in primates^5^, even after correcting for phylogenetic relatedness^3^. (II) Parrots are geographically widespread and can live in different places around the world, such as the Austral conure (living in the southern hemisphere below 54°S (Tierra del Fuego) and the Alexandrine Parakeet (*Psittacula eupatria)* occurring northerly at around 33°N (Afghanistan^37^. Similarly, they occur at different altitudes, with some (e.g. *Psilopsiagon aurifrons*) being sighted even at 4500 m. As such parrots allow for testing hypotheses that suggest ecological functions for external eye appearance. Finally, III) there is variation in social systems within this taxonomic group, specifically regarding breeding systems, sexual dimorphism, and flock size, allowing the examination of social hypotheses. Therefore, in the present study, we carry out a preliminary investigation of the potential effect of allometric scaling rules, and variables of ecological and social nature on the external eye appearance of Psittaciformes.

### Hypotheses

With regards to allometric scaling rules, we first expected heavier parrots to have more exposed eyeballs (Sclera Surface Index: SSI). While we expect SSI to increase with mass, this relationship may be less pronounced than in primates due to the lighter skeleton of birds^21–23^.

Regarding ecological factors, we investigated whether external eye appearance is related to typical species’ ranges by using latitude and maximum living altitude. Latitude and altitude influence a multitude of abiotic factors. On average, there are greater amounts of visible light and UV radiation found closer to the equator^38,39^ and at higher altitudes^40,41^. Latitude is closely linked to humidity and temperature, which in turn may affect the proliferation of bacteria^12^. Altitude is also directly related to temperature, and sky turbidity^42^, and has been shown to affect color vision^43,44^, but see^45^. Pigmentation can absorb excess visible light and UV radiation, thus preventing damage to tissue and it may also regulate the amount and quality of light entering the retina, enhancing visual perception^29,46^. In individuals with albinism, the lack of pigments close to the pupil results in increased glare (ocular straylight^47^). Therefore, we expected to find darker irises closer to the equator and at higher altitudes (cf.^3^). Because the thickness of the troposphere differs depending on latitude (being thickest at the equator and thinning with distance from it), we also added an interaction term in our model. To more specifically test whether UV radiation may be affecting the tissues we measured, we also regressed previously published estimations of UV-B incidence^19^ in our species’ distribution with our measurements. Lastly, we investigated whether the inner irises of the species in our sample reflected more green-blue light with increasing distance from the equator. This was found to be the case in primates^3^, suggesting an adaptation to compensate for lower levels of blue light, important for circadian regulation, in greater latitudes.

Regarding social functions, we investigated potential links between species’ breeding system, flock size and conspicuity of the eye, expecting that cooperatively breeding species would display greater differences in brightness between parts of the eye. Contrast between pupil and iris could enhance the perception of changes in pupil size, which has been shown to regulate processes of physiological synchrony in humans and other apes^48^, and to mediate the perception of trust in humans^49,50^. During social dominance interactions and courtship displays, parrots are engaged in constriction of the pupil which is called ‘eye-pinning’^34,35^. One study shows that rapid pupil constriction occurs in synchrony with vocalizations in a parrot (*Amazona ochrocephala panamensis*), suggesting that rapid changes in pupil size may be part of a multimodal complex of audiovisual displays^51^.

We also tested whether chromatic salience of the eyes of sexually dimorphic species was greater than species where both sexes have a similar physical appearance. Although not as common as differences in plumage, sexual dimorphism in eye color has also been reported for birds^26^, and among parrots, the iris does vary between the sexes in some species (e.g., Cacatua species^35^). Because of parrots’ propensity to engage in eye-pinning in social signaling, the eye could be of special importance for social signaling functions. We expect the inner and outer part of the irises to be more different in hue in sexually dimorphic species as this difference could act as an attention-getter and could contribute towards perceiving salient morphs in conjunction with other tissues that are known to signal sex in dichromatic species (like feathers).

Lastly, we investigated whether greater differences in brightness between the pupil and the inner part of the iris, and between the inner and the outer part of the iris were related to flock size. Contrast between adjacent tissues of the eyeball has been extensively studied in primates due to the possibility that conspicuous eyeballs could inform conspecifics about an individual’s focus of attention^1–3,5,52^, Therefore, this ocular contrast could help in assessing the focus of attention of neighboring individuals, to facilitate flocking behavior. Thus, we test whether, as proposed in primates, differences in brightness in adjacent parts of the eye could be related to behaviors requiring spatial coordination between conspecifics^53^.

## Results

We measured the eyes of 4–5 individuals from 132 parrot species, resulting in a total of 647 individuals sampled. Due to time constraints, we selected these species randomly. Full details of the sampling strategy can be found in the Methods section. All results are summarized below in Table 1. For each species in our sample, we calculated the mean and standard deviation for all measurements, including: pupil brightness; brightness and hue of the inner and outer parts of the iris; surrounding skin hue and brightness. Mean values for inner and outer iris brightness and hue are mapped on the phylogeny in Figures S1–S4, which also shows an estimation of the ancestral states. Some of our independent variables were not available for all species. The actual number of species included is indicated for each analysis. When models did not meet parametric assumptions, we transformed the dependent variable with Tukey’s ladder of power.

**Table 1:** Summary of results from the models above. Coefficients, and 95% CI for PGLS analyses, and F for phylogenetic ANOVAs. P values in phylogenetic ANOVAs are based on simulations, hence no df is reported.

	Sclera size index (SSI)	Inner iris brightness (Tuk. transf.)	Inner iris brightness (Tuk. transf.)	Outer iris brightness (Tuk. transf.)	Outer iris brightness (Tuk. transf.)	Brightness of tissue surrounding the eye (Tuk. transf.)	Brightness of tissue surrounding the eye (Tuk. transf.)	Inner iris hue (Tuk. transf.)	Brightness difference between inner & outer iris (Tuk. transf.)	Brightness difference between pupil & inner iris (Tuk. transf.)	Hue difference between inner & outer iris (Tuk. transf.)
Intercept	1.060*** (1.035, 1.085)	2.024*** (1.853, 2.195)	1.726*** (1.519, 1.932)	5.651*** (4.400, 6.903)	3.787*** (2.739, 4.835)	1.897*** (1.778, 2.015)	1.853*** (1.638, 2.069)	−0.242*** (−0.304, −0.180)	1.619***(1.230, 2.008)	2.100*** (1.891, 2.309)	
Mass (KGs)	0.004 (−0.055, 0.063)										
Latitude (absolute, in ºs)		−0.017*** (−0.029, −0.004)		−0.048 (−0.133, 0.036)		−0.006 (−0.015, 0.002)		0.0003 (−0.001, 0.002)			
Max living altitude (KMs)		−0.120***(−0.203, −0.037)		−0.323 (−0.921, 0.274)		−0.034 (−0.093, 0.025)					
Latitude:max living altitude		0.009*** (0.003, 0.015)		0.020 (−0.024, 0.063)		0.003 (−0.001, 0.008)					
Flock size									0.0002 (−0.0003, 0.001)	0.0002 (−0.001, 0.001)	
Breeding system									F = 0.93	F = 0.79	
Sexual dimorphism									F = 0.38	F = 0.81	F = 0.38
UV-B			0.00000 (−0.00004, 0.00005)		0.00003 (−0.0002, 0.0002)		0.00004 (−0.00004, 0.00005)				

Significance: *p < 0.1; **p < 0.05; ***p < 0.01.

To test whether body mass affects the amount of exposed eyeball, we regressed the amount of exposed eyeball (measured as sclera surface index or SSI; cf.^5^^,^^54^) over species’ average weight but we found no statistical relationship (β = 0.0039298, SE = 0.02994500, t = 0.13123, p = 0.8961, n = 53; Figure S5 in the supplementary materials).

**Figure 2:**
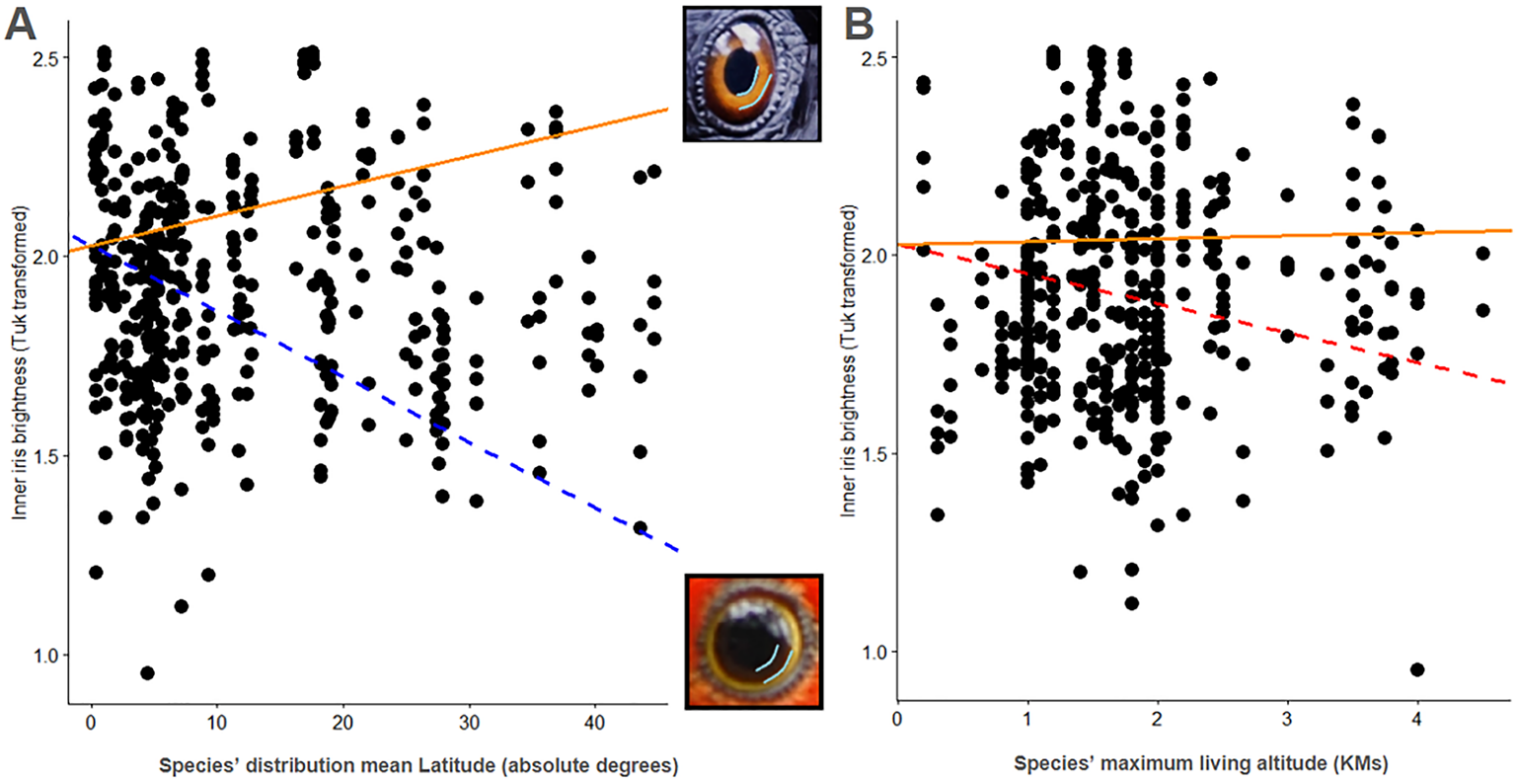
The results of our (**A**) Brightness of the inner iris regressed over mean latitude of our species’ distribution and (**B**) maximum living altitude. The dependent variable was transformed with Tukey’s ladder of powers (Tuk transformed); see Methods. Solid regression lines depict the interaction between Latitude and Altitude. The blue dashed line represents the main effect of Latitude and the red dashed line represents the main effect of altitude in our model. The inner portion of the iris is darker in species further from the equator, and in species with higher living altitude. This is not the case in species that both have ranges far from the equator, and high living altitude. The inner part of the iris is highlighted in cyan for two species representing the higher (*Chalcopsitta atra*) and lower (*Alisterus scapularis*) values in our dataset. Original photo credits: Schristia and latch.r, CC BY 2.0 https://creativecommons.org/licenses/by/2.0.

To test whether the brightness of different parts of the eye was related to environmental factors, we regressed the brightness of these parts of the eye over mean latitude of the species’ distribution, as well as over the maximum living altitude recorded for each of our species. The inner part of the irises (the portion of the iris closest to the pupil) are significantly darker farther away from the equator (β = −0.0166342, SE = 0.00620356, t = −2.681403, p = 0.0076, n = 92; Fig. 2). The negative association between inner iris brightness and species’ maximum living altitude was also significant (β = −0.1199481, SE = 0.04242098, t = −2.827565, p = 0.0049, n = 92). A significant interaction of latitude and maximum living altitude indicated that, in species living in higher altitude, there was an increase (instead of decrease) of brightness of the inner part of the iris associated with distance from the equator, though the effect was small in comparison to the main effects (β = 0.0088866, SE = 0.00317970, t = 2.794779, p = 0.0054, n = 92; Fig. 2), and may be best interpreted as a lack of reduction in the brightness of the inner part of the iris in species that both live far from the equator, and at high altitudes. To elucidate whether the effects of latitude could be related to UV-B radiation (e.g. specifically driven by photo-protective functions), we ran another PGLS with inner iris regressed over UV-B measurements at the centroids of the distribution of the species in our sample, but found no association (β = 0.0000047, SE = 0.00002107, t = 0.221049, p = 0.8251, n = 119). We also tested the effects of UV-B on the brightness of the outer iris, and surrounding tissue but found no effect (outer iris: β = 0.000033, SE = 0.0001061, t = 0.308993, p = 0.7574, n = 119; surrounding tissues: β = 0.0000361, SE = 0.00002211, t = 1.631037, p = 0.1034, n = 121). Unlike the inner part of the iris, no association was found either between brightness of the outer part of the iris and latitude (β = −0.048454, SE = 0.0432790, t = −1.119577, p = 0.2635, n = 92), maximum living altitude (β = −0.323379, SE = 0.3047095, t = −1.061269, p = 0.2892, n = 92), nor their interaction (β = 0.019527, SE = 0.0222803, t = 0.876430, p = 0.3813, n = 92). Likewise, we found no association between the brightness of the tissue surrounding the eye and latitude (β = −0.0064744, SE = 0.00439702, t = −1.472460, p = 0.1416, n = 92), maximum living altitude (β = −0.0340355, SE = 0.03013762, t = 1.129337, p = 0.2594, n = 92), nor their interaction (β = 0.0031613, SE = 0.00225445, t = 1.402253, p = 0.1616, n = 92). Lastly, we tested whether the hue of the inner part of the iris increased with distance from the equator (shifting from reflecting more red-yellow, to more green–blue light), but found no association (β = 0.00026411, SE = 0.000844875, t = 0.312603, p = 0.7547, n = 120).

We investigated whether species with a potentially greater selective pressure for communicating with ocular displays (as in eye pinning) had greater contrast between the inner part of the iris and the pupil, as a way to facilitate the perception of changes in pupil size. We ran a phylogenetic ANOVA with the difference in brightness between pupil and inner part of the iris as the dependent variable and breeding system as independent variable. We found no significant differences when comparing between species living in colonies, those cooperatively breeding, monogamous, or polyandrous species (F = 0.785226, p = 0.556). The difference in brightness between inner and outer part of the iris could also facilitate the perception of changes in pupil size, since the thickness of both inner and outer part of the iris changes substantially when pupils are entirely dilated or constricted. Therefore, we tested whether different breeding systems displayed variation in the difference in measurements of brightness between inner and outer part of the iris, but again found no significant difference (F = 0.924615, p = 0.46, n = 39). We also used a phylogenetic ANOVA to test whether differences in hue between the inner and outer part of the iris were a feature of sexually dimorphic species, but there was again no significant difference between sexually dimorphic and monomorphic species in our sample (F = 0.381749, p = 0.823, n = 62). Differences in brightness between the pupil and inner part of the iris were assessed across sexually monomorphic and dimorphic species by means of another phylogenetic ANOVA, but there were no differences (F = 0.80793, p = 0.738, n = 62). Lastly, we tested whether conspicuous eyes could serve as visual cues to facilitate spatial dynamics by allowing the inference of other individuals’ focus of attention. To do so, we ran two PGLS analyses. In one, we regressed the difference in brightness between inner and outer part of the iris over flock size. In the other, we regressed the difference in brightness between the inner part of the iris and pupil over flock size. We did not find an association between differences in brightness of inner and outer part of the iris, and flock size (β = 0.0002282, SE = 0.00028843, t = 0.791208, p = 0.4295, n = 64), nor between differences in brightness between inner part of the iris and pupil, and over flock size (β = 0.0001572, SE = 0.00041153, t = 0.381893, p = 0.7028, n = 64).

## Discussion

By testing associations between characteristics of external eye appearance and species-level anatomical data, spatial distribution, and social behavior, we found evidence supporting ecological functions, but not social functions, of external eye morphology in Psittaciformes. We found no significant relationship between a greater amount of exposed eyeball (SSI) and body mass, in contrast to previous results in primates^3,54^. Regarding ecological hypotheses, we found specifically that the portion of the iris closer to the pupil (which we termed “inner part of the iris”) tends to be darker in species with their distributions farther from the equator, and in species with greater living altitude. This decrease in the inner part of the iris brightness was less pronounced in species with greater living altitude that were also distributed farther from the equator. However, we found no effect of UV-B measurements in the same tissue. This suggests that abiotic factors that co-vary with latitude and altitude, other than UV-B radiation, drive the variation we observed. We discuss the potential effects of visible light in driving this cline in more detail below.

Our results did not show greater SSI with increased body mass, in contrast with previous findings in primates^3,55^. Birds have lighter heads and flexible necks, making it possible for them to scan horizontally by moving their heads in addition to their eyes (representing a characteristic behavior in birds called head saccades^55^. Moving the head provides more stability to fixations than eye movements^56^, benefitting foraging and reducing predation risk. The allometric relationship between eye and mass previously reported in primates may be different in birds, who have larger eyes per unit of body mass compared to mammals and whose bones are lighter than those of mammals^57–60^. Furthermore, even though birds have been shown to move their eyeballs to reorient their attention^24^, they also bob their entire heads, similar to how the smallest primate species may do so to, for example, better estimate distances^61^. Finally, although parrots show a relatively large variation in body sizes in comparison to some other avian orders, this does not necessarily compare to the variation observed in for example primates, and the head mass of the heaviest parrot in our sample may simply not be heavy enough to favor eye movement over head movement. Therefore, it would be particularly interesting to study the potential effect of this allometric scaling rules in taxonomic groups that also include much heavier birds (e.g. *Palaeognathae*), or in birds with particularly large bills (e.g. *Ciconiiformes*, *Ardei*, or *Bucerotidae)*.

PGLS analyses showed that darker inner parts of the irises (the portion of the iris closer to the pupil) were associated with species ranging farther from the equator, and with greater maximum living altitude. Previous investigations suggested that pigmentation around the pupil has a protective role in regulating the amount of light passing through the iris opening and reducing the glare in different avian groups^29^. Species distributed far from the equator may have a darker inner part of their irises to prevent glare (the anti-glare hypothesis^14^ by absorbing stray light before it enters the retina through the opening in the iris). Dusk and dawn are characterized by an increase in scattered light and are substantially longer in greater latitudes as the summer solstice approaches. Evidence from humans also shows that visual sensitivity is greatest during dusk and dawn^62^, which could be an ancestral adaptation of land vertebrates also affecting other tetrapod lineages to optimize vision during conditions of low light. If this were the case, momentary orientation towards the sun could result in intense glare in parrots as well. While we also found that the interaction between latitude and maximum living altitude resulted in a lighter inner part of their irises, this effect was not as substantial as the effect of maximum living altitude (which was one order of magnitude greater), or the effect of latitude (which was two orders of magnitude greater). Thus, the interaction between altitude and latitude may be better interpreted as a reduction of the main effects of latitude and altitude on the inner part of the iris brightness when both co-vary positively.

While our results suggest that the photopic conditions typical of species affect their eye appearance, the effect we found is small, and in the opposite direction from what has been found in previous studies. This warrants caution in interpreting these results as biologically meaningful and make it unlikely that our results reflect specifically photoprotective adaptations. Photoprotection in birds has been proposed for various tissues^19,63^, but our results specifically show that our measurements are not related to UV-B. In addition, the variation we observe (darker farther from the equator) is the opposite one would expect for photoprotective needs, as UV radiation from the sun is strongest at the equator. At the infraorder level (anthropoid primates)^3^, tested possible associations between social and ecological variables, and different measurements of external eye appearance. Like^2^, they found that on a group level, eye appearance was not associated with any social variables. Instead, pigmentation around the iris, and iris color were associated with the latitude of the centroid of the distribution of species. This led the authors to propose that local differences in lighting drove species’ level external eye appearance. It is crucial to factor in how different species relate to their photopic environments.

While our results suggest that parrot external eye appearance is influenced by photopic factors, the affected tissue (inner portion of the iris) and the direction of the effect (darker with increasing distance from the equator) are different than in primates. In primates, the tissue affected is the conjunctiva, around the iris, and the tissue becomes darker (not lighter) closer to the equator^3^. In primates, the darkening of the conjunctiva has been proposed to comply with photoprotective functions, safeguarding repositories of epithelial stem cells that are needed to keep the cornea transparent^15^. In primates, thus, ocular tissues may have co-evolved to comply with the same photoprotective functions as hair and skin color. This may not be the case in birds, who can have markedly different colors in different integumentary tissues (e.g. black skin and white feathers^19^). As our SSI measurements suggest, the corneal limbus (the interstice between the cornea and surrounding tissues) of a parrot is typically covered by skin. We are not aware of any sensitive population of cells in the inner irises. Because the inner iris is the edge of the pupil, which lets light into the retina we speculate that our findings relate to visual perception, similar to the functions proposed by^29^ in buttonquails. The additional analyses showing no relationship between UV-B and inner iris brightness also lead us to think in this direction. However, this explanation remains speculative until other sources of evidence can complement our results—for example, histological examination of the tissues, or functional studies on vision in different species or luminosity conditions. In this regard, it is important to note that satellite UV-B measurements are a useful tool for macroecological studies but are not without limitations—they can differ from ground readings^64–67^, especially for readings at high altitudes due to atmospheric phenomena such as extinction rate of airmass, water vapor, and aerosol^68^. These readings are also taken at 15-arc minute intervals^69^, which can constitute a rather coarse interval. Lastly, our imputation method may introduce random noise because the UV-B estimates closest to the centroids of our species’ ranges may not be necessarily representative of irradiation throughout the species’ distribution. It would have been preferable to obtain an integrated average estimate across the entire distribution of the species,

In contrast to our results with latitude and altitude, we found no evidence for any of the social variables we included in our tests to be related to the external eye appearance of the parrots. Therefore, our study provides preliminary evidence that the function of external eye morphology in Psittaciformes is related to ecological factors, rather than to social factors. While these results do not preclude that the external eye appearance of specific species of parrot may have evolved under selection pressures for signaling, they do suggest that, at least at the order level, the selection pressures that shape external eye appearance are more basal and likely related to differences in their photopic environments.

Our results may appear to contrast with studies that have found external eye appearance to be related to social variables in birds (e.g.^9^) and primates^4^. In the case of studies with birds, it should be noted that these studies have identified social functions when focused on a single species; for example, Ref^9^ found that jackdaws rely on ocular conspicuity to deter conspecifics from entering their nests, but the same function was not found for e.g. Passeriformes^28^. Behavioral traits are more plastic than morphological traits^70^. As a result, closely related species may share morphological traits (i.e. external eye appearance) but differ in their social behavior. Large scale studies including broad phylogenies are therefore more likely to find associations with global factors. This is also in line with^8^’s conclusion that ecological variables are more likely to predict iris color, compared to signaling (“social”) functions. When it comes to primates, one study shows a correlation between levels of scleral pigmentation and intraspecific lethal aggression^4^; by contrast, see^2,3,25^ recently compared a sample of domesticated species with their wildtype relatives and found no evidence that peri-iridal depigmentation was more prevalent in domesticates, undermining the idea that peri-iridal depigmentation is related to temperament (including lethal aggression) in wild species. While the neural-crest cell hypothesis^71^ predicts overall losses of pigmentation in self-domesticated species, the mechanism whereby conspecific aggression leads to greater pigmentation levels is not mentioned in^4^. In short, while there is some evidence linking external eye appearance in birds to social factors, this becomes evident only at the species level. Lastly, it should be noted that our results could still be related to social factors – if light conditions are such that darker colors become more conspicuous at higher latitudes and altitudes.

To conclude, the present study provides evidence suggesting that environmental factors, possibly related to light, affect the external eye appearance of parrots. It should be noted that, compared to previously published studies that quantify external eye appearance using the current methods, our study has less samples per species (~ 5, compared to ~ 20 in^3^). Our null results should thus be taken with caution, as they may be due to a lack of power from reduced sample size. While the present study informs our growing understanding of the influence of light in external eye appearance in terrestrial vertebrates, it also suggests that the relationship between light and external eye appearance is mediated by other factors such as behavior. Furthermore, our sampling strategy was comprehensive in the number of species but included relatively few samples, so random conditions of lighting in the photos may have blurred out effects. Previous investigations using uncalibrated photographs estimated a minimum of 12 samples for measurements consistent with museum specimens^72^. While previous studies have relied on photographs to subjectively score iridal coloration^8,28^ ours is the first to report on chromatic qualities (i.e., hue), which should make us cautious about using them as ground-truth in subsequent studies. This is especially important to note in our measurements of (inner and outer) iris hue, in which intra-species variance exceeds inter-species variance (Table S3). Future studies could focus on narrower taxa (genus and below), or functionally defined groups (e.g. arboreal vs. land living) in which the relationship between patterns of appearance and biological functions may be discernible, as well as including larger sample size per species. While considering bird vision was out of the scope of the present investigation, it could be beneficial for subsequent studies to take into consideration known aspects of bird vision such as visual field^73,74^) or color perception^75^. This approach has been previously applied to specific primate species^76^, largely corroborating the results of early investigations using our approach. However, this should only be done taking into consideration differences in visual systems between species.

## Methods

### Taxonomic samples

All the photographs we measured come from publicly available photographs on the Internet (n = 647). To find photographs of a given species, we entered the full name in binomial nomenclature. If any contextual information on the website could suggest that this was not the species of interest, we did not include it in the sample. This could include the exclusive use of a vulgar name or mentioning only the genus. If the photographed animal deviated substantially from other individuals we found of the same species, we excluded that sample. The full list of photographs we used, together with the links to their sources is available via the link under *Data availability*. Data were collected between June 2020 and March 2022 by ED, DMJP, CV, and JJMM. We included photographs in our sample only if the different parts of the eye were distinguishable. We avoided photographs that were obviously edited, over, or underexposed. Our samples included 132 Psittaciformes species. We aimed to include two random species per genus. Sometimes this was not possible due to lack of materials. In that case we either found the next most derived species in the taxon or left that branch (representing that genus) with only one tip if no more species were available. We aimed to collect at least 5 photographs of different adult individuals per species but, in some cases, this was not possible (mean number of photos per species = 4.45 range = 1–5). We identified individuals whenever possible to avoid sampling the same one twice. When it was not clear whether the animal had already been sampled, we omitted the photograph.

### Eye measurements

**Figure 3:**
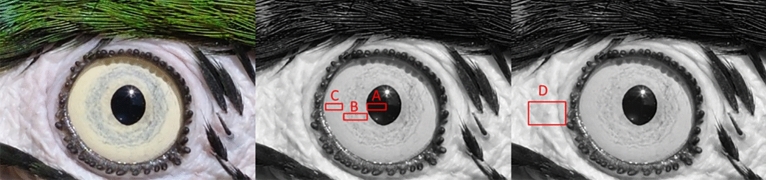
The eye of Ara ararauna. (**A**) is the sampling area of pupil brightness, (**B**) is the sampling area of the inner part of the iris hue and brightness, (**C**) is the sampling area of the outer part of the iris hue and brightness, and (**D**) stands for the sampling area of tissue surrounding the eye hue and brightness. Original photo credits: Quartl, CC BY-SA 3.0 https://creativecommons.org/licenses/by-sa/3.0/deed.en.

For each photograph, we sampled the eye on the left of the photograph. If that eye showed confounding factors (stark shadows, specular reflections), or was not pictured laterally for eye shape measurements, we used the right eye. We measured our images with the PAT-GEOM, an ImageJ add-on permitting color and brightness measurements in HSB^77^. We measured sclera size index (SSI)^20^, brightness and hue of the inner and outer part of the iris, as well as the tissue immediately adjacent to the eye (typically featherless skin; Fig. 3). SSI measurements are obtained by dividing the width of the exposed eyeball by the width of the iris, resulting in a ratio. SSI was measured only in photographs without obvious lid closure, and in which one eye was facing the camera frontally (e.g. face was laterally exposed). All distances were measured in pixels. The iris was measured from the edge of the pupil towards the edge of the iris, or before reaching confounding factors such as specular reflections, skin or feathers, etc. From the measurements of brightness, we calculated the difference in measurements of brightness between adjacent tissues: inner part of the iris-pupil, inner-outer part of the iris, outer part of the iris-surrounding tissue. We opted for absolute differences between parts of the eye because of the issues Caspar et al.^2^ and Mearing & Koops^78^ raised regarding ratio-based measurements (like RIL). We measured iris hue in degrees (Hue Saturation Brightness or HSB^79^) with PAT-GEOM’s HSB-ColorMeasure tool^77^. Measurements of surrounding skin were not included in any analyses. Species-level data are available in the supplementary materials.

### Phylogenetic data, anatomic, social, and ecological variables

We used a consensus tree built from a combination of molecular data from^80^ to account for phylogeny in our PGLS and phylogenetic ANOVAs. Maximum living altitude (e.g. the highest above sea level the species have been observed living) and latitude of the centroid of species’ distribution, and social variables (flock size, breeding system, and whether species were considered sexually dimorphic or not) were taken from a combination of^81^, iNaturalist, and previously published species’ level data^82,83^. The breeding systems included in our data set are Colonial, Cooperative, Monogamous, and Polyandrous. Flock sizes may change depending on the season. In our data, we annotated the maximal observed flock size. The maximum living altitude was coded in kilometers. We used multiple sources to incorporate variables that were missing from other sources. When two sources contradicted each other, we used the most recent source. Species level measurements of mass were taken from^36^. For our PGLS with UV-B as an independent variable, we used the data from^69^. First, we used QGIS to draw the shape of the distribution of each species and obtain the centroid of their distribution. Then, we matched the coordinates to the closest datapoint in^69^. When the centroid of an irregularly shaped distribution fell clearly outside the shape, we split it in two and used the average value corresponding to both centroids. When a species’ distribution was split into several landmasses, we took the centroid of the landmass where the species was originally from, and disregarded distributions for which the species was known to be invasive. If the species’ range was split in several land masses, but the species was native to all those (e.g. in archipelagos), we averaged the UV-B values corresponding to the centroids of each landmass.

### Statistics and reproducibility

Because phenotypic traits are influenced not only by adaptation, but also by past evolutionary history, we used phylogenetic generalized least squares (PGLS) and phylogenetic ANOVA—methods that incorporate information about phylogenetic relatedness to account for non-independence of data points between the species in our sample^84^. Ocular traits (SSI, Brightness of the inner and outer part of the iris, inner and outer part of the iris hue, and average difference in measurements of brightness between inner and outer part of the iris) were used as response variables in separate regression models (PGLS) or phylogenetic ANOVAs. Body mass, average latitude of the species’ distribution, breeding system, sexual dimorphism, and maximum living altitude were used as independent variables. For the latitude data, we first made the values absolute to express distance from the equator. For PGLS, we examined diagnostic plots, and applied transformation to variables if the transformation improved the homoscedasticity of residuals. Transformations included Tukey transformation and log. For phylogenetic ANOVAs, we tested the residuals of response variables for normality with Shapiro–Wilk tests, and applied Tuk transformation when variables were not normally distributed. The two nocturnal species in our sample (*Pezoporus occidentalis* and *Strigops habroptilus*) were removed from analyses relating eye traits with photopic drivers (iris hue and inner and outer part of the iris brightness over distance from the equator). Average inter-rater reliability (IRR) was calculated for ~ 5% of the measurements (n = 35 photographs) at 0.914 for Pupil Brightness, 0.877 for inner part of the iris Hue, 0.903 for inner part of the iris Brightness, 0.943 for outer part of the iris Hue, 0.992 for outer part of the iris Brightness, 0.608 for the hue of the tissue surrounding the eye, and 0.669 for the brightness of the tissue surrounding the eye. Only photographs for which both ED and JOPG could agree that they were fully lateral were included in the sample for SSI measurements. Fully lateral was defined as photographs taken from one side of the animal so that only one side of their body was visible. Photographs in which both nostrils or eyes were visible were excluded from this category. Only 55 photographs were used for SSI measurements, so the entire dataset was included in the IRR calculations. For all our SSI measurements, the IRR was 0.841. Values from both raters were averaged and used in the analyses. Phylogenetic analyses vary in the number of species, depending on available data for each variable and species, as well as whether they were present in our phylogenetic tree block. All analyses were conducted in R (4.3.2)^85^. For phylogenetic ANOVA we used the phylANOVA function from the phytools R package^86^. We used the pgls function with Pagel’s *lambda* correlation structure^87^ from the caper package in R^88^ for our PGLS analyses. We assumed no intraspecific geographic variation in this study. Such variation was not readily apparent in our sample-individuals of a single species tend to have similar external eye appearance. In general, this seems to be the case in most avian species^89^. We believe it is likely that there are differences across sexes and development, but this was not the focus of our study. Previous studies reported sex and age differences. Some of these studies are in parrots^35^. We ran additional analyses excluding species previously reported to be dimorphic in ocular traits. The results were qualitatively identical. These analyses are reported in the supplementary materials (table S2). Phylograms in the supplementary materials were built with the plotTree function from the package phytools.

### Supplementary Information

Supplementary Information.

## Data Availability

Links to the photographs, raw measurements, variables included in the study, and the code used for the analyses can be found in the following link: https://osf.io/9hxvm/?view_only=12e3021c24c74a6ca20437f0d99e8da4.
