# Supplementary material for: Preliminary evidence that eye appearance in parrots (Psittaciformes) co-varies with latitude and altitude
Source: Sci Rep. 2024 Jun 4;14:12859. doi: 10.1038/s41598-024-63599-3 (PMC11150271; doi:10.1038/s41598-024-63599-3)
Supplement: Supplementary file 1 — Supplementary Information. [file 41598_2024_63599_MOESM1_ESM.docx]

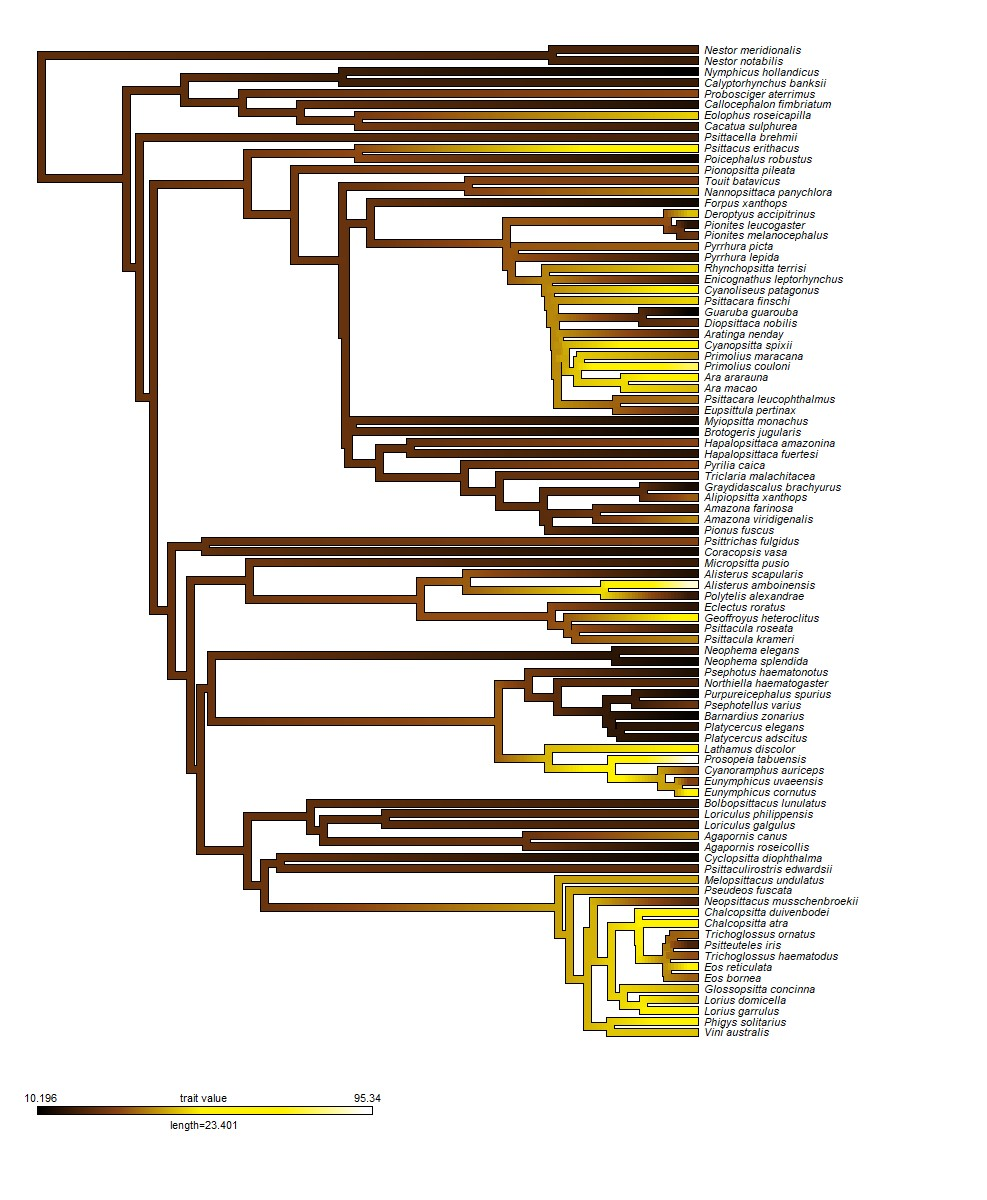
Figure S1.- Inner iris brightness mapped over the parrot phylogeny. Values represent darker to lightest.

Figure S2.- Outer iris brightness mapped over the parrot phylogeny. Values represent darker to lightest
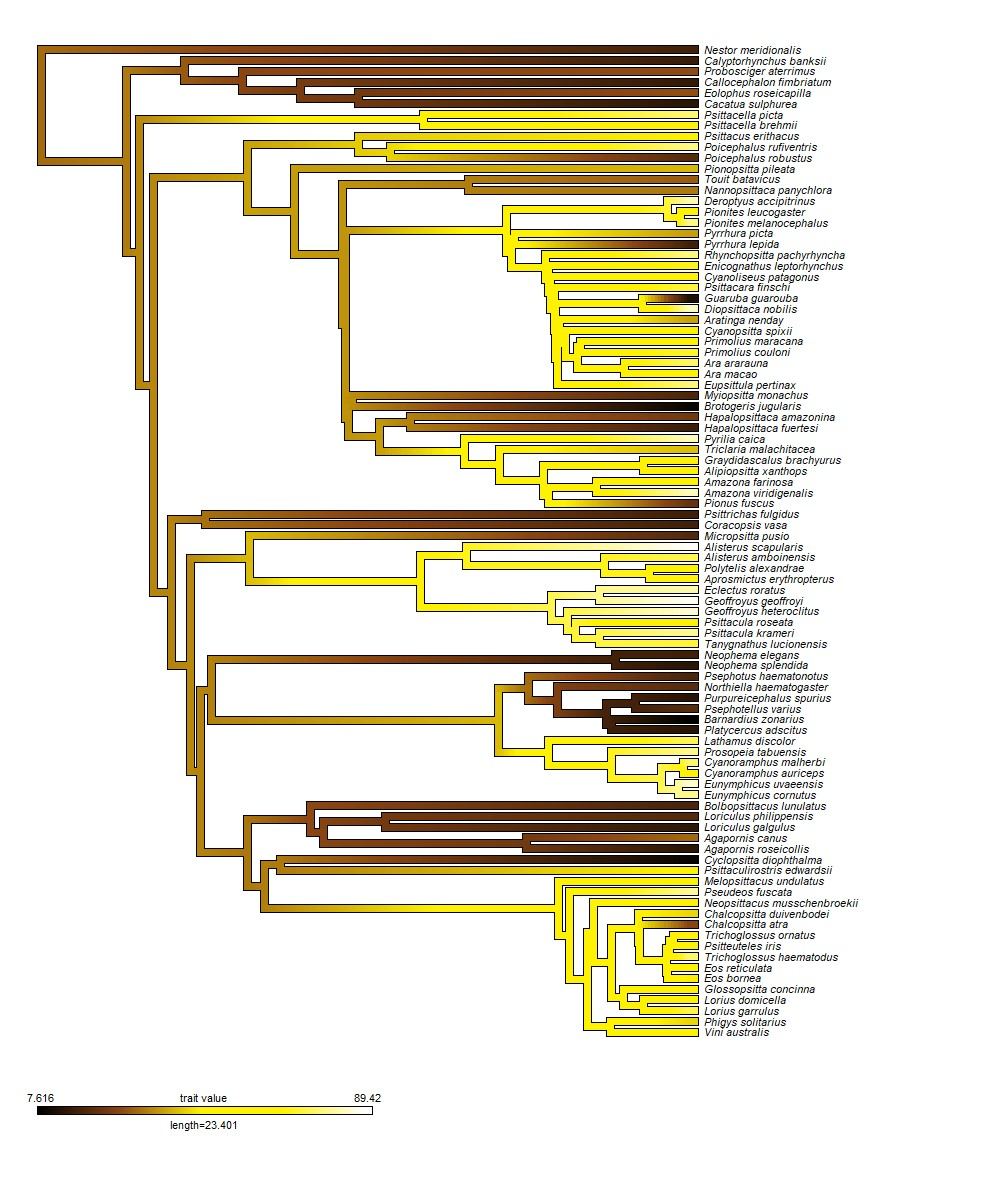


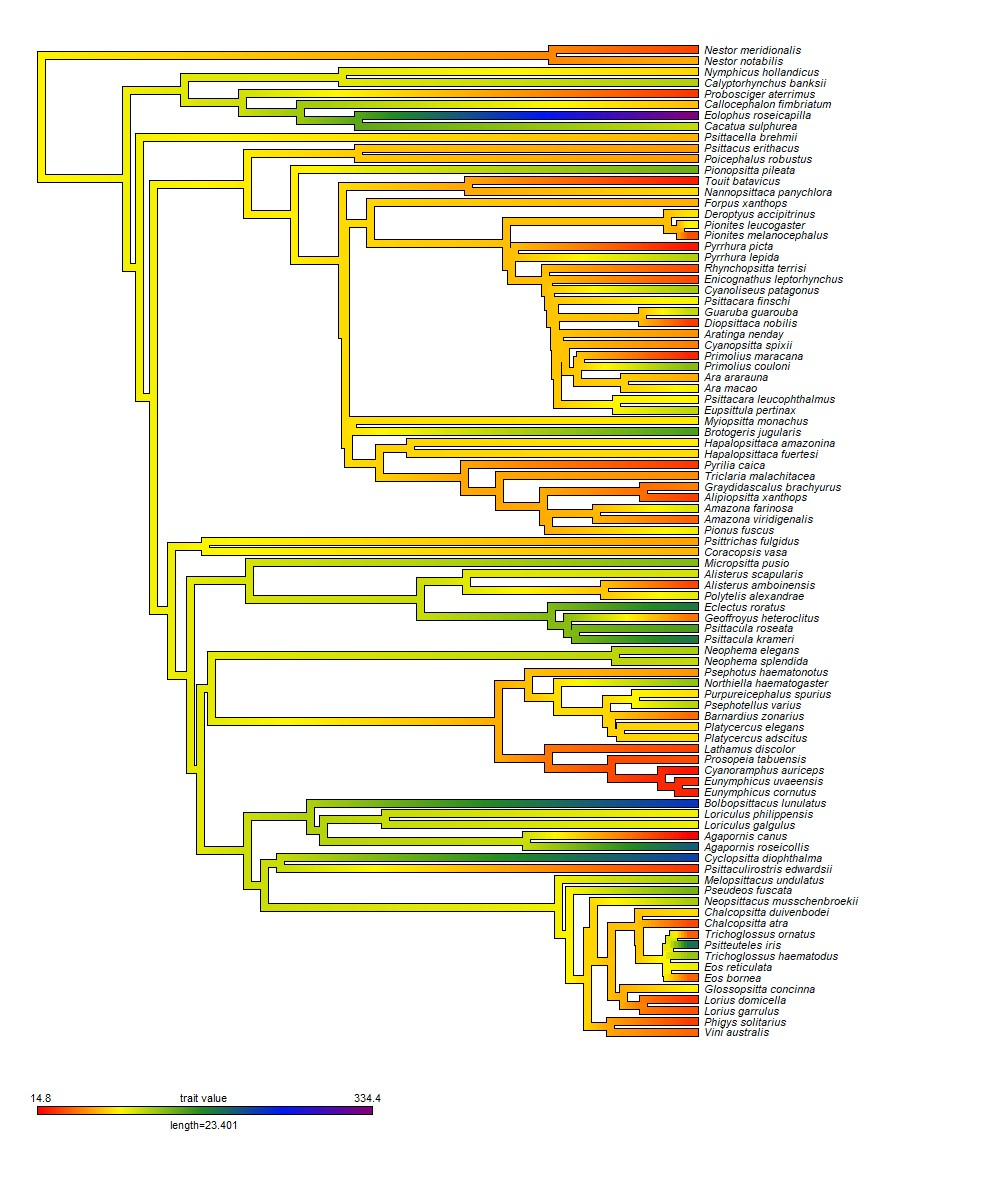
Figure S3.- Inner iris hue mapped over the parrot phylogeny. Colors in the hue trees approximate actual measurements.


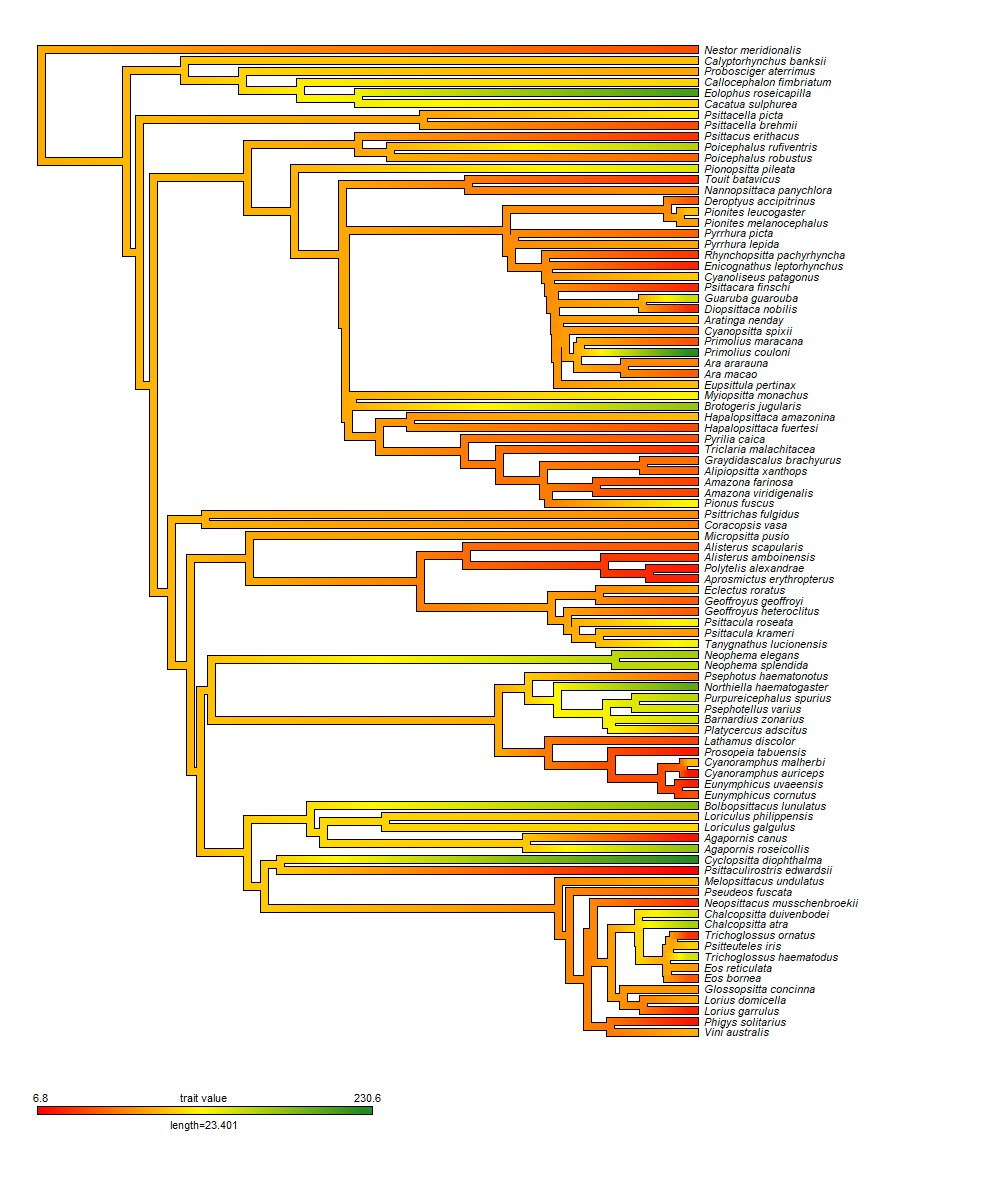
Figure S4.- Outer iris hue mapped over the parrot phylogeny. Colors in the hue trees approximate actual measurements


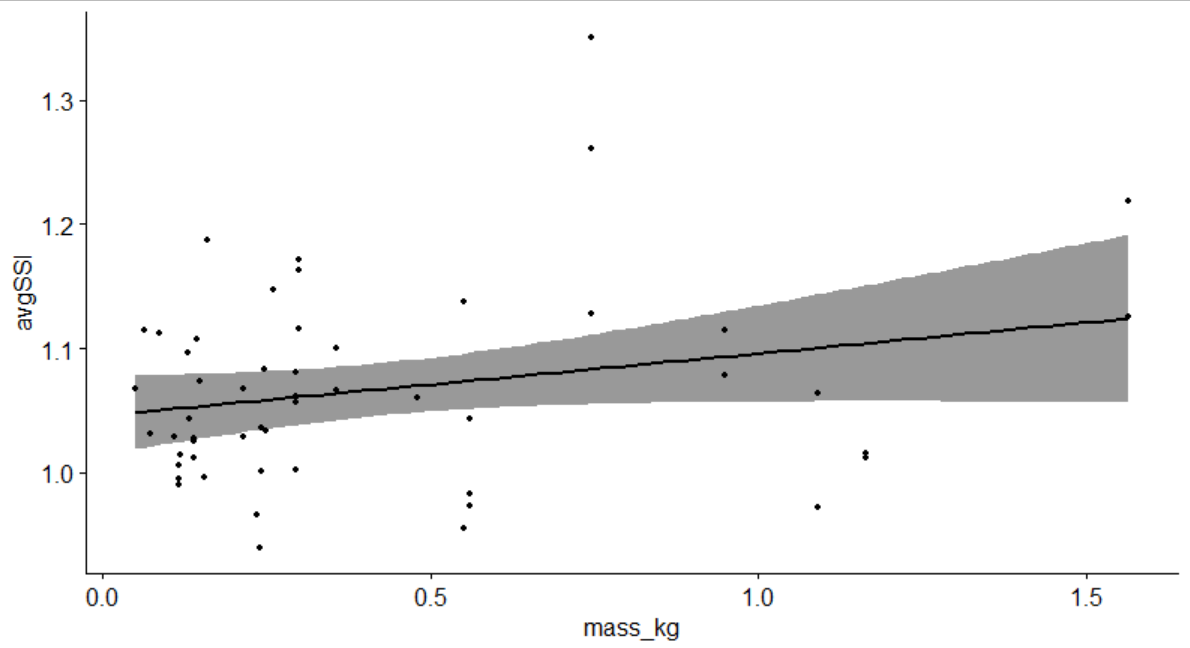


*Figure S5.-* The relationship between exposure of the eyeball (Sclera Surface Index or SSI) and body mass in kilograms.

Table S1.- Summary of measurements per species.

| **Species** | **PupilBleft.mean** | **InnerIrisB.mean** | **OuterIrisB.mean** | **SurroundingB.mean** | [**InnerIrisB.sd**](http://inneririsb.sd) | [**OuterIrisB.sd**](http://outeririsb.sd) | [**SurroundingB.sd**](http://surroundingb.sd) | **InnerIrisH.mean** | **OuterIrisH.mean** | **SurroundingH.mean** | [**InnerIrisH.sd**](http://innerirish.sd) | [**OuterIrisH.sd**](http://outerirish.sd) | [**SurroundingH.sd**](http://surroundingh.sd) |
| --- | --- | --- | --- | --- | --- | --- | --- | --- | --- | --- | --- | --- | --- |
| Agapornis_canus | 5.116667 | 38.8712 | 32.3924 | 24.3698 | 11.01147 | 9.59048 | 7.047683 | 14.8 | 14 | 63.6 | 8.043631 | 5.244044 | 55.89097 |
| Agapornis_roseicollis | 4.5022 | 14.7412 | 13.971 | 11.1466 | 5.304065 | 5.490095 | 1.723132 | 207 | 175.8 | 193.4 | 130.267 | 141.1177 | 121.8885 |
| Alipiopsitta_xanthops | 7.1372 | 34.5644 | 59.394 | 28.3158 | 13.70845 | 23.37055 | 9.676581 | 35 | 51.2 | 69.6 | 4.743416 | 63.33798 | 25.47155 |
| Alisterus_amboinensis | 5.7708 | 92.5462 | 75.407 | 38.4118 | 11.32904 | 16.45915 | 10.72124 | 36.6 | 32.4 | 198.6 | 11.43678 | 11.7601 | 145.0941 |
| Alisterus_scapularis | 10.2705 | 15.4966 | 89.4202 | 30.9338 | 10.30513 | 12.78255 | 8.083542 | 110.4 | 45.8 | 128.8 | 119.6591 | 7.224957 | 57.84203 |
| Amazona_farinosa | 8.8824 | 19.912 | 65.6386 | 26.9446 | 18.9887 | 13.98305 | 13.48323 | 107 | 36 | 124.6 | 105.7473 | 42.35564 | 61.60195 |
| Amazona_viridigenalis | 6.65 | 39.0982 | 84.464 | 37.7988 | 32.4783 | 14.17311 | 12.30838 | 46.2 | 37.2 | 104.6 | 13.36787 | 12.69646 | 24.01666 |
| Anodorhynchus_hyacinthinus | 9.9498 | 5.8635 | 6.15875 | 4.93925 | 2.862743 | 3.16721 | 2.416481 | 78.25 | 131.25 | 132.75 | 67.25263 | 83.48802 | 63.67299 |
| Anodorhynchus_leari | 5.426667 | 12.53475 | 9.78475 | 10.399 | 8.040549 | 6.206815 | 6.657283 | 189.75 | 119 | 170.4 | 96.77594 | 84.53402 | 87.04769 |
| Aprosmictus_erythropterus | 11.77567 | 25.81975 | 69.1922 | 39.1634 | 8.070781 | 15.89345 | 9.089661 | 27.75 | 22 | 49 | 10.96586 | 5.744563 | 15.57241 |
| Aprosmictus_jonquillaceus | 10.8684 | 60.397 | NA | 52.5405 | 24.98915 | NA | 30.16871 | 21.5 | NA | 82 | 4.949747 | NA | 19.79899 |
| Ara_ararauna | 6.5345 | 57.5978 | 77.3574 | 79.5906 | 17.21518 | 10.88146 | 7.249779 | 70.7 | 67.5 | 116.9 | 69.87298 | 66.39319 | 107.4031 |
| Ara_macao | 4.96525 | 46.121 | 60.61483 | 73.62117 | 14.73088 | 13.34679 | 17.33742 | 97.83333 | 48.83333 | 135.1667 | 58.95563 | 5.56477 | 97.29628 |
| Aratinga_jandaya | 11.4325 | 33.32533 | 35.867 | 18.353 | 7.97403 | 13.42235 | 12.17722 | 95.33333 | 109.3333 | 199.4 | 121.8373 | 134.0386 | 102.5271 |
| Aratinga_nenday | 8.835 | 23.0052 | 38.84146 | 19.9966 | 13.69942 | 18.40967 | 9.428649 | 62.2 | 83.2 | 118.6 | 60.79638 | 147.6743 | 28.68449 |
| Barnardius_zonarius | 8.656 | 11.2764 | 7.6156 | 24.7856 | 5.998044 | 7.740914 | 10.20207 | 49.2 | 141 | 249.8 | 58.19966 | 76.64529 | 29.54996 |
| Bolbopsittacus_lunulatus | 4.1048 | 19.4002 | 18.398 | 24.5874 | 13.92178 | 14.89954 | 8.400879 | 234.6 | 180.2 | 214.6 | 117.6873 | 129.2002 | 76.68637 |
| Bolborhynchus_ferrugineifrons | 8.60225 | 12.004 | NA | 52.418 | 15.86748 | NA | 1.895046 | 99.5 | NA | 62 | 72.832 | NA | 19.79899 |
| Bolborhynchus_lineola | 5.378667 | 15.625 | 20.06775 | 10.703 | 8.937374 | 15.46372 | 3.710512 | 31.25 | 89.25 | 128.4 | 18.22773 | 129.8393 | 93.42537 |
| Brotogeris_jugularis | 12.3968 | 11.2222 | 8.8692 | 8.3126 | 6.970796 | 6.302 | 5.39832 | 160.4 | 171.8 | 185 | 139.2921 | 117.1269 | 86.42627 |
| Brotogeris_tirica | 6.6955 | 18.162 | 19.755 | 68.869 | 7.590094 | 10.32437 | 13.99413 | 79 | 72.5 | 169.75 | 124.3248 | 101.0099 | 77.62034 |
| Cacatua_goffiniana | 5.2655 | 11.5425 | 13.247 | 9.0215 | 6.94369 | 7.081948 | 5.168311 | 217.25 | 238.25 | 175.5 | 161.3493 | 134.1228 | 115.4484 |
| Cacatua_sulphurea | 4.37925 | 19.3872 | 13.7492 | 74.8496 | 12.3731 | 3.889095 | 13.28153 | 116 | 103.2 | 217 | 99.01768 | 100.5967 | 17.81853 |
| Callocephalon_fimbriatum | 15.6816 | 15.298 | 15.3218 | 9.9402 | 7.802179 | 12.12002 | 4.299498 | 77.2 | 104 | 115 | 82.953 | 116.6726 | 73.5629 |
| Calyptorhynchus_banksii | 8.3205 | 19.1974 | 16.0652 | 16.2968 | 4.311633 | 4.880044 | 5.064699 | 122.8 | 93.2 | 141.8 | 155.983 | 104.1499 | 92.38073 |
| Chalcopsitta_atra | 11.38083 | 74.3926 | 27.2674 | 44.026 | 14.92216 | 8.792842 | 15.86082 | 36.8 | 164 | 164.8 | 9.257429 | 137.6572 | 100.7135 |
| Chalcopsitta_duivenbodei | 7.4132 | 61.3414 | 45.3594 | 17.4314 | 22.74757 | 15.68618 | 6.319783 | 86.6 | 144.2 | 256.4 | 84.67762 | 194.7324 | 54.78412 |
| Charmosyna_papou | 6.3506 | 26.652 | 74.6035 | 35.339 | 2.868025 | 12.55185 | 4.647106 | 171.5 | 24 | 122.5 | 190.2117 | 1.414214 | 101.1163 |
| Charmosyna_placentis | 16.5226 | 32.63267 | 78.84967 | 30.05 | 4.562629 | 12.72145 | 3.806366 | 36.33333 | 15 | 70.33333 | 25.54082 | 9.165151 | 47.71094 |
| Coracopsis_nigra | 4.7799 | 12.1325 | 16.721 | 84.1385 | 5.01268 | 11.72383 | 8.36578 | 23.5 | 19 | 72.5 | 3.535534 | 5.656854 | 50.20458 |
| Coracopsis_vasa | 5.1332 | 13.6336 | 17.1162 | 12.5416 | 8.980883 | 11.63736 | 8.189412 | 73.6 | 65.2 | 107 | 81.22376 | 100.2731 | 79.38829 |
| Cyanoliseus_patagonus | 6.6732 | 55.44317 | 52.81367 | 31.61033 | 17.4434 | 14.24727 | 6.953527 | 128.5 | 99.5 | 142.6667 | 89.39743 | 91.54835 | 93.19156 |
| Cyanopsitta_spixii | 9.3765 | 63.6382 | 52.6726 | 40.476 | 7.356528 | 5.009159 | 7.44936 | 55.6 | 58 | 105.8 | 42.15804 | 12 | 28.03034 |
| Cyanoramphus_auriceps | 12.95433 | 33.9722 | 59.5316 | 32.055 | 17.97803 | 17.75225 | 14.75009 | 20.6 | 9.4 | 162.4 | 16.69731 | 8.203658 | 114.3517 |
| Cyanoramphus_malherbi | 6.330667 | 17.93633 | 78.8404 | 29.1436 | 2.426188 | 11.52922 | 13.47391 | 23.33333 | 96.8 | 186.2 | 3.511885 | 147.2573 | 60.04332 |
| Cyclopsitta_diophthalma | 18.695 | 12.099 | 9.0516 | 23.948 | 2.795373 | 5.633051 | 1.334051 | 223.2 | 230.6 | 233 | 89.91496 | 95.75907 | 43.70927 |
| Cyclopsitta_gulielmitertii | 7.05875 | 14.10125 | 13.67975 | 22.441 | 9.643854 | 10.29905 | 10.09229 | 152.75 | 155.25 | 176 | 156.9806 | 159.5501 | 99.7831 |
| Deroptyus_accipitrinus | NA | 47.4376 | 84.1396 | 46.1532 | 12.91509 | 16.58054 | 7.42452 | 89.2 | 44.2 | 125.2 | 67.19152 | 14.82228 | 38.02236 |
| Diopsittaca_nobilis | 12.7068 | 25.4902 | 85.641 | 34.8996 | 9.128793 | 13.75214 | 9.828799 | 33.2 | 24.6 | 109.4 | 11.90378 | 20.69541 | 37.95787 |
| Eclectus_roratus | 9.7902 | 17.21775 | 82.72 | 46.106 | 8.213198 | 13.35597 | 24.3149 | 187.125 | 79.125 | 163.375 | 94.13886 | 78.60287 | 116.7475 |
| Enicognathus_ferrugineus | 12.925 | 30.5145 | 22.3385 | 29.78575 | 15.37345 | 7.902893 | 11.85978 | 27.75 | 18.25 | 74.25 | 5.315073 | 7.973916 | 100.1911 |
| Enicognathus_leptorhynchus | 3.92425 | 20.8654 | 70.8998 | 24.159 | 7.411969 | 22.90026 | 8.92896 | 38.2 | 22.6 | 88.2 | 25.09382 | 11.28273 | 34.96713 |
| Eolophus_roseicapilla | 8.1456 | 48.46 | 29.2616 | 53.4242 | 30.10619 | 16.90785 | 13.35515 | 334.4 | 212.4 | 282.2 | 36.37032 | 191.2206 | 104.4447 |
| Eos_bornea | 4.9244 | 34.4204 | 47.625 | 29.3722 | 8.474383 | 15.4115 | 10.37077 | 42.8 | 44.2 | 105.2 | 24.01458 | 75.96841 | 14.9566 |
| Eos_reticulata | 8.22675 | 54.1962 | 51.2 | 39.6958 | 24.0584 | 28.3434 | 15.96808 | 102.6 | 76.2 | 210.6 | 118.652 | 147.5676 | 87.09937 |
| Eunymphicus_cornutus | 7.934 | 56.1052 | 79.9726 | 31.2744 | 17.71093 | 9.765335 | 5.342954 | 25.8 | 44.6 | 91 | 9.284396 | 67.5633 | 68.64037 |
| Eunymphicus_uvaeensis | 12.6092 | 28.3356 | 86.04 | 19.5946 | 16.5688 | 12.72924 | 10.15018 | 29.8 | 17.4 | 152.2 | 13.27403 | 8.648699 | 85.58446 |
| Eupsittula_nana | 4.2488 | 53.9215 | 57.549 | 85.50675 | 16.38196 | 22.20073 | 15.97263 | 19 | 21.5 | 55.25 | 5.477226 | 8.504901 | 16.39868 |
| Eupsittula_pertinax | 6.7618 | 25.8338 | 78.7208 | 27.6494 | 6.583265 | 7.119784 | 5.789175 | 118.2 | 94 | 143.2 | 74.52986 | 135.3754 | 43.61422 |
| Forpus_passerinus | 6.9668 | 21.555 | 17.0935 | 11.81625 | 11.06459 | 8.346672 | 6.342281 | 27.5 | 25.5 | 46 | 7.234178 | 9.398581 | 18.86796 |
| Forpus_xanthops | 11.0152 | 13.1604 | 19.2465 | 50.2194 | 11.33958 | 11.62739 | 21.11667 | 72.2 | 62.25 | 173.4 | 58.84046 | 49.33812 | 145.0424 |
| Geoffroyus_geoffroyi | 9.9604 | 37.4355 | 88.6882 | 41.3188 | 8.569419 | 12.11575 | 9.914236 | 58.25 | 48.4 | 71.4 | 25.224 | 8.443933 | 36.7192 |
| Geoffroyus_heteroclitus | 4.091333 | 55.7614 | 86.0918 | 55.6414 | 15.75577 | 11.23689 | 7.60392 | 51.4 | 47.4 | 60.2 | 10.50238 | 9.787747 | 29.09811 |
| Glossopsitta_concinna | 21.21733 | 44.9488 | 54.1812 | 22.7744 | 17.12291 | 19.93765 | 10.59621 | 93.4 | 75 | 157.4 | 88.49181 | 127.648 | 90.20976 |
| Graydidascalus_brachyurus | 6.7202 | 13.8708 | 49.3964 | 21.0748 | 4.807166 | 9.884306 | 8.4548 | 55.6 | 58.6 | 75.2 | 35.21789 | 63.74402 | 53.11026 |
| Guaruba_guarouba | 15.5814 | 10.9858 | 10.81 | 9.5918 | 7.116888 | 2.946127 | 3.869198 | 117.2 | 148.8 | 155.4 | 94.51296 | 135.9162 | 50.53019 |
| Hapalopsittaca_amazonina | 11.5322 | 30.7856 | 24.3334 | 18.4808 | 9.86016 | 7.876915 | 6.747419 | 91.6 | 92.4 | 142.6 | 98.4571 | 99.66093 | 90.17372 |
| Hapalopsittaca_fuertesi | 7.9348 | 16.7384 | 16.3398 | 12.4818 | 7.854938 | 6.69298 | 5.312216 | 86.2 | 40.2 | 90.4 | 59.7386 | 8.642916 | 38.39661 |
| Lathamus_discolor | 3.3436 | 53.5268 | 75.7246 | 34.5954 | 28.09311 | 23.63682 | 14.08164 | 35.8 | 36.4 | 85.4 | 3.03315 | 5.504544 | 57.38728 |
| Leptosittaca_branickii | 6.9872 | 21.1465 | 31.27525 | 19.1605 | 7.534499 | 14.81032 | 8.119239 | 232.75 | 253.5 | 225.25 | 132.5654 | 163.5043 | 99.82109 |
| Loriculus_galgulus | 9.026 | 20.29 | 14.6762 | 13.0542 | 6.59166 | 4.355231 | 3.798162 | 101.2 | 104.4 | 136.4 | 106.4786 | 98.76133 | 81.65354 |
| Loriculus_philippensis | 4.617 | 23.2182 | 20.1076 | 17.7562 | 2.702623 | 6.192443 | 3.94751 | 99.2 | 90.2 | 112.4 | 79.0993 | 106.4411 | 55.74316 |
| Lorius_domicella | 6.7944 | 45.572 | 52.5876 | 28.4746 | 14.19439 | 26.08432 | 8.90314 | 30.6 | 83.4 | 135.6 | 6.9857 | 131.8893 | 56.78292 |
| Lorius_garrulus | 8.6744 | 64.8234 | 75.2288 | 45.5866 | 12.04389 | 21.39291 | 23.83817 | 40.6 | 22.6 | 149.2 | 7.127412 | 3.911521 | 131.5112 |
| Melopsittacus_undulatus | 9.4142 | 43.007 | 64.8616 | 31.6274 | 31.53068 | 13.34104 | 15.16896 | 129.6 | 87 | 101 | 80.61203 | 90.61733 | 75.39562 |
| Micropsitta_finschii | 5.794 | 38.235 | 41.66667 | 27.83233 | 18.1909 | 16.13289 | 6.90689 | 67.66667 | 48.33333 | 57.66667 | 80.83522 | 53.46338 | 29.48446 |
| Micropsitta_pusio | 10.2818 | 18.9218 | 19.9868 | 46.1038 | 5.938271 | 12.1524 | 3.952606 | 139.8 | 69.4 | 86.8 | 161.1605 | 116.5989 | 125.84 |
| Myiopsitta_luchsi | NA | NA | NA | NA | NA | NA | NA | NA | NA | NA | NA | NA | NA |
| Myiopsitta_monachus | 3.22125 | 14.1084 | 19.032 | 12.6098 | 7.028039 | 8.167683 | 6.652431 | 90.6 | 120.4 | 143.8 | 101.8519 | 82.26968 | 78.4965 |
| Nannopsittaca_dachilleae | 8.2556 | 21.59475 | 30.637 | 15.00975 | 10.1554 | 18.66511 | 5.669923 | 165 | 219 | 197.25 | 140.9704 | 149.6151 | 113.9777 |
| Nannopsittaca_panychlora | 14.2866 | 39.883 | 34.1502 | 24.8858 | 18.18511 | 11.50135 | 8.233637 | 85.8 | 70.8 | 134.8 | 144.46 | 123.7 | 105.6229 |
| Neophema_elegans | 6.4596 | 19.5782 | 17.0848 | 14.6084 | 12.37108 | 12.32798 | 11.19333 | 124.8 | 165.2 | 162.8 | 87.17626 | 90.12047 | 63.76284 |
| Neophema_splendida | 9.0984 | 12.0644 | 13.7728 | 27.4734 | 5.183808 | 3.478182 | 38.29209 | 116.2 | 149.6 | 131.4 | 135.6252 | 154.0221 | 102.4246 |
| Neopsephotus_bourkii | 6.7724 | 11.43175 | 12.66025 | 10.956 | 3.011955 | 5.097911 | 4.32476 | 237.75 | 146 | 205.25 | 107.6952 | 139.018 | 65.74889 |
| Neopsittacus_musschenbroekii | 12.89375 | 23.2938 | 48.8824 | 24.6854 | 21.87135 | 30.06445 | 15.0325 | 126.8 | 30.6 | 135.8 | 95.35041 | 38.61735 | 49.32748 |
| Neopsittacus_pullicauda | 5.7886 | 34.353 | 83.137 | 28.707 | NA | NA | NA | 211 | 18 | 218 | NA | NA | NA |
| Nestor_meridionalis | 7.996 | 22.3348 | 17.494 | 16.7704 | 9.352264 | 9.968795 | 5.427284 | 36.6 | 41.8 | 85.2 | 23.96456 | 29.34621 | 23.08029 |
| Nestor_notabilis | 13.3316 | 19.4514 | 13.8345 | 50.249 | 18.7421 | 8.479726 | 30.87633 | 71 | 145 | 40.8 | 128.9729 | 161.2514 | 6.016644 |
| Northiella_haematogaster | 9.9088 | 22.7604 | 20.4772 | 40.2942 | 10.3438 | 12.19307 | 5.252461 | 134.6 | 201 | 188.6 | 166.3169 | 123.2599 | 129.6256 |
| Nymphicus_hollandicus | 3.6588 | 10.19633 | 11.09425 | 50.66433 | 6.956851 | 3.222821 | 15.19426 | 84.66667 | 18.25 | 61.5 | 107.2001 | 6.800735 | 92.79386 |
| Oreopsittacus_arfaki | 16.6102 | 21.25 | 20.669 | 28.576 | 15.08662 | 11.7906 | 16.52571 | 100.5 | 176.5 | 150.6 | 119.5729 | 173.2907 | 110.0695 |
| Orthopsittaca_manilatus | 12.7302 | 24.32925 | 20.3405 | 16.03325 | 7.421703 | 10.82655 | 9.767536 | 31.5 | 57.5 | 70.5 | 19.75686 | 30.0943 | 46.50806 |
| Pezoporus_wallicus | 6.5772 | 45.77033 | 47.83933 | 29.394 | 23.30545 | 26.62341 | 9.694931 | 88.66667 | 55.33333 | 118.3333 | 78.29006 | 15.17674 | 57.72637 |
| Phigys_solitarius | 9.2018 | 57.072 | 45 | 23.268 | 26.62836 | 32.50597 | 6.171692 | 33.6 | 15.4 | 139.6 | 6.024948 | 1.516575 | 124.102 |
| Pionites_leucogaster | 4.7432 | 15.3878 | 52.8852 | 20.7468 | 5.517764 | 3.6187 | 6.254473 | 99.8 | 96.2 | 108.4 | 109.2071 | 149.6302 | 53.48177 |
| Pionites_melanocephalus | 5.94275 | 26.8816 | 81.0984 | 33.8062 | 2.967338 | 16.23277 | 12.71521 | 35.8 | 79 | 100.6 | 11.43241 | 95.9036 | 45.70886 |
| Pionopsitta_pileata | 7.19375 | 36.5868 | 41.6604 | 21.4514 | 23.50222 | 24.8194 | 10.69315 | 148.6 | 136.4 | 150.2 | 121.5249 | 126.2549 | 118.8621 |
| Pionus_fuscus | 9.9224 | 13.5524 | 22.6492 | 16.4828 | 8.85996 | 20.15923 | 9.485421 | 99.6 | 120 | 110.6 | 76.97597 | 82.17968 | 71.95693 |
| Pionus_menstruus | 11.336 | 17.99675 | 13.069 | 12.10225 | 8.467173 | 6.48182 | 6.497342 | 71.25 | 115.5 | 130 | 67.72678 | 93.56103 | 39.37004 |
| Platycercus_adscitus | 2.565 | 13.5784 | 13.5426 | 28.2746 | 5.626882 | 5.195483 | 11.94406 | 84.6 | 80.2 | 127 | 87.17396 | 116.7399 | 109.5719 |
| Platycercus_elegans | 5.059 | 16.666 | 15.18775 | 14.115 | 7.555961 | 7.854199 | 6.929538 | 83.8 | 86.25 | 109.6 | 106.8162 | 82.02591 | 70.26592 |
| Poicephalus_robustus | 10.1114 | 12.5532 | 20.0072 | 11.67675 | 6.411795 | 14.29755 | 5.612142 | 65 | 51.6 | 58.25 | 51.5558 | 39.09348 | 54.67099 |
| Poicephalus_rufiventris | 10.9464 | 45.435 | 79.805 | 34.6734 | 11.81789 | 19.82648 | 12.28798 | 155 | 157.6 | 192.2 | 157.734 | 157.2142 | 79.83232 |
| Polytelis_alexandrae | 5.8266 | 17.761 | 68.42933 | 57.6155 | 7.496478 | 8.019668 | 5.958215 | 103.3333 | 18.33333 | 128.1667 | 134.4807 | 4.366539 | 124.0765 |
| Polytelis_swainsonii | 11.0326 | 14.7205 | 68.427 | 25.253 | 10.72469 | 24.86768 | 17.22718 | 107.5 | 36 | 123.75 | 109.6016 | 7.348469 | 49.30433 |
| Primolius_couloni | 9.1154 | 82.2388 | 72.9472 | 52.5502 | 22.48748 | 21.45083 | 4.042781 | 139.8 | 230.6 | 175.2 | 67.59956 | 128.3094 | 29.77751 |
| Primolius_maracana | 6.7035 | 41.4878 | 61.0186 | 34.2664 | 19.02767 | 10.42209 | 6.363769 | 26 | 43.8 | 80.6 | 6.557439 | 66.69858 | 22.18783 |
| Prioniturus_discurus | 7.1774 | 10.122 | 9.974 | 59.2865 | 3.160767 | 2.670035 | 34.6546 | 140.5 | 157 | 193 | 41.7193 | 31.1127 | 36.76955 |
| Prioniturus_platurus | 6.9356 | NA | NA | NA | NA | NA | NA | NA | NA | NA | NA | NA | NA |
| Probosciger_aterrimus | 8.2076 | 31.843 | 28.7286 | 35.2772 | 9.939534 | 10.26289 | 17.60989 | 32.4 | 81.2 | 181.2 | 32.46229 | 145.8259 | 130.0219 |
| Prosopeia_personata | 7.26075 | 97.64233 | 85.52833 | 67.51267 | 2.312413 | 11.34154 | 6.483165 | 30.66667 | 60.66667 | 72.33333 | 8.621678 | 81.69659 | 46.30695 |
| Prosopeia_tabuensis | NA | 95.3398 | 80.2634 | 32.1636 | 3.694234 | 18.31358 | 12.10903 | 38.2 | 17.6 | 111.2 | 16.99117 | 3.361547 | 99.08431 |
| Psephotellus_varius | NA | 26.5634 | 20.7222 | 38.7356 | 7.251683 | 8.236704 | 9.12094 | 121.4 | 138.8 | 117.8 | 139.1467 | 155.384 | 88.77894 |
| Psephotus_haematonotus | 10.1114 | 15.2076 | 18.5188 | 11.692 | 3.987479 | 6.233293 | 2.851038 | 64.2 | 57 | 74.6 | 53.49019 | 47.58151 | 43.44307 |
| Pseudeos_fuscata | 10.974 | 38.48163 | 81.52325 | 35.24388 | 23.31764 | 15.39835 | 23.65725 | 145.75 | 50 | 212.875 | 104.4793 | 125.3452 | 98.98981 |
| Psilopsiagon_aurifrons | 14.2038 | 27.131 | 29.567 | 15.12267 | 7.124808 | 10.75368 | 7.166219 | 25 | 25.5 | 55 | 1.414214 | 0.707107 | 49.38623 |
| Psilopsiagon_aymara | 6.9654 | NA | NA | NA | NA | NA | NA | NA | NA | NA | NA | NA | NA |
| Psittacara_finschi | 11.2745 | 49.1486 | 73.5814 | 32.6374 | 8.422295 | 9.521874 | 6.533512 | 98.4 | 24.6 | 99.2 | 83.41942 | 4.878524 | 40.34476 |
| Psittacara_leucophthalmus | 8.9726 | 36.9744 | 67.68575 | 31.8454 | 14.75301 | 8.925571 | 13.37071 | 94.6 | 90.75 | 136.2 | 93.86853 | 152.2594 | 89.56953 |
| Psittacella_brehmii | 4.0164 | 20.9105 | 66.271 | 35.1795 | 5.417053 | 20.93885 | 31.38017 | 73.33333 | 39 | 98.33333 | 66.76426 | 70.24528 | 42.25005 |
| Psittacella_picta | 8.7104 | 25.71025 | 78.2732 | 27.7912 | 8.651458 | 21.94477 | 5.417405 | 246.25 | 112.4 | 170 | 157.6861 | 141.9834 | 56.75826 |
| Psittacula_krameri | 5.9108 | 39.4564 | 81.0892 | 33.3936 | 3.955864 | 19.25926 | 6.744333 | 188.4 | 77.6 | 147.2 | 46.44674 | 118.913 | 74.25429 |
| Psittacula_roseata | 10.847 | 18.3372 | 50.9288 | 17.7426 | 6.099108 | 27.9039 | 8.320592 | 160.6 | 122.8 | 150.4 | 98.29191 | 122.5488 | 107.1975 |
| Psittaculirostris_desmarestii | 8.74675 | 29.8555 | 32.84325 | 21.70925 | 24.1777 | 18.82188 | 15.69173 | 62 | 87 | 76.25 | 63.1981 | 139.3676 | 47.24669 |
| Psittaculirostris_edwardsii | 7.5466 | 23.8594 | 49.392 | 41.6208 | 11.92941 | 13.66013 | 9.742723 | 31.6 | 6.8 | 141.2 | 23.41581 | 2.588436 | 100.7308 |
| Psittacus_erithacus | 9.5094 | 62.⁶ | 48.59283 | 63.08 | 16.53913 | 8.260496 | 11.79979 | 64.66667 | 29.16667 | 161 | 57.5071 | 12.35179 | 95.72878 |
| Psittacus_timneh | 7.5746 | 73.82125 | 67.519 | 41.92825 | 14.00971 | 9.406269 | 5.87157 | 90.25 | 65.75 | 146.25 | 83.56784 | 60.91182 | 50.95341 |
| Psitteuteles_iris | 9.0174 | 20.7268 | 60.5198 | 24.9668 | 9.655108 | 19.40133 | 6.124201 | 202.4 | 101.4 | 170.8 | 116.6503 | 138.1079 | 95.84727 |
| Psitteuteles_versicolor | 4.8104 | 19.41725 | 31.77125 | 84.0925 | 11.5234 | 12.86904 | 6.110356 | 129.5 | 79.25 | 148.75 | 124.7411 | 78.90659 | 69.00423 |
| Psittrichas_fulgidus | 14.1896 | 30.2696 | 16.8188 | 33.9402 | 12.43764 | 5.60597 | 16.29436 | 66.8 | 70.4 | 134 | 87.90165 | 92.21876 | 125.6045 |
| Purpureicephalus_spurius | 5.7148 | 13.3392 | 13.4668 | 28.7196 | 6.283932 | 5.853334 | 15.04794 | 87.8 | 154.2 | 171.6 | 93.60128 | 120.5454 | 126.243 |
| Pyrilia_caica | 4.6696 | 31.9168 | 84.3798 | 36.0074 | 12.1359 | 13.1953 | 5.509013 | 32.2 | 44 | 86.6 | 5.932959 | 60.14566 | 49.983 |
| Pyrilia_haematotis | NA | 41.52475 | 46.4005 | 93.83875 | 18.24437 | 11.37375 | 4.706305 | 107.5 | 79.5 | 183 | 74.46476 | 91.19028 | 27.43477 |
| Pyrrhura_lepida | 9.5955 | 16.8564 | 17.3378 | 92.5964 | 3.803676 | 6.644497 | 4.795563 | 123 | 81.6 | 172.2 | 132.3329 | 72.9678 | 61.12037 |
| Pyrrhura_picta | 8.9652 | 34.325 | 38.5148 | 21.7556 | 23.60648 | 25.85139 | 12.65374 | 21.6 | 52 | 70.4 | 8.792042 | 59.3675 | 47.4426 |
| Rhynchopsitta_pachyrhyncha | 9.873667 | 49.10417 | 79.32543 | 43.69429 | 21.3051 | 10.33906 | 13.49308 | 45.5 | 31.71429 | 82.71429 | 18.26198 | 10.60997 | 24.01884 |
| Rhynchopsitta_terrisi | 14.8348 | 48.8432 | 66.03725 | 39.4305 | 15.66017 | 15.60878 | 9.290254 | 38.6 | 21 | 73.5 | 18.1604 | 6.928203 | 27.73085 |
| Tanygnathus_lucionensis | 14.2035 | 20.5815 | 73.3136 | 54.479 | 6.551276 | 25.34422 | 10.41879 | 141.25 | 124 | 114.6 | 100.2044 | 103.0024 | 70.65975 |
| Tanygnathus_sumatranus | 15.905 | NA | NA | NA | NA | NA | NA | NA | NA | NA | NA | NA | NA |
| Touit_batavicus | 7.5238 | 28.9282 | 30.7712 | 15.9622 | 17.29582 | 14.65776 | 9.106665 | 23.4 | 27.8 | 99.6 | 8.648699 | 8.258329 | 85.04587 |
| Touit_purpuratus | 9.0524 | NA | NA | NA | NA | NA | NA | NA | NA | NA | NA | NA | NA |
| Trichoglossus_haematodus | 3.162 | 32.111 | 78.8142 | 36.5948 | 8.471507 | 26.07349 | 23.73753 | 134 | 145.4 | 174.8 | 95.77317 | 193.182 | 120.0696 |
| Trichoglossus_ornatus | 9.6664 | 36.0832 | 61.986 | 43.7852 | 26.10308 | 29.34588 | 23.0586 | 40.4 | 24 | 41.8 | 32.25368 | 39.19821 | 17.3407 |
| Triclaria_malachitacea | 5.1568 | 25.664 | 42.3302 | 28.4928 | 13.04508 | 13.76614 | 8.248718 | 60.6 | 28 | 103.8 | 45.46207 | 7.648529 | 68.39371 |
| Vini_australis | 13.831 | 47.1274 | 57.245 | 49.8868 | 24.87229 | 27.36932 | 25.52123 | 47.6 | 87.8 | 96.6 | 28.91021 | 149.5466 | 83.53323 |

Table S2.- Results for models using sexual dimorphism as a predictor variable without species previously reported to present sexually dimorphic eye appearanc: *Cacatua goffiniana, Cacatua sulphurea, Calyptorhynchus banksii, Nymphicus hollandicus,* and *Probosciger aterrimus.* F for phylogenetic ANOVAs. P values in phylogenetic ANOVAs are based on simulations, hence no df is reported. Significance: *p<0.1; **p<0.05; ***p<0.01.

|  | *Brightness difference between pupil & inner iris (Tuk. transf.)* | *Hue difference between inner & outer iris (Tuk. transf.)* |
| --- | --- | --- |
| *Sexual dimorphism* | F=2.5 | F=0.39 |

Table S3.- Comparisons of Inter- and Intra-species variance in our measurements of inner and outer iris brightness and hue, and measurements of brightness of the tissue surrounding the eye.

|  | *Inner iris brightness* | *Outer iris brightness* | *Inner iris hue* | *Outer iris hue* | *Brightness of tissues surrounding the eye* |
| --- | --- | --- | --- | --- | --- |
| *Intra-species variance* | 207.2999 | 247.2985 | 6945.608 | 8685.469 | 160.8358 |
| *Inter-species variance* | 355.9067 | 701.9823 | 3602.267 | 3168.777 | 351.6261 |
